# Comparative analysis of 5-year efficacy and outcomes of single anastomosis procedures as revisional surgery for weight regain following sleeve gastrectomy

**DOI:** 10.1007/s00464-023-10234-3

**Published:** 2023-07-11

**Authors:** Asaad F. Salama, Jawher Baazaoui, Fakhar Shahid, Rajvir Singh, Antonio J. Torres, Moataz M. Bashah

**Affiliations:** 1 Bariatric and Metabolic Surgery Department, Hamad General Hospital (HGH), Hamad Medical Corporation (HMC), Doha, Qatar; 2 Research Center, Heart Hospital, Hamad Medical Corporation (HMC), Doha, Qatar; 3 Department of Surgery, Complutense University of Madrid, Hospital Clinico “San Carlos”, Madrid, Spain; 4 Department of Surgery, Weill Cornell Medical College, Doha, Qatar

**Keywords:** Revisional surgery, Weight regain, Sleeve gastrectomy, Single anastomosis duodeno-ileal bypass, One anastomosis gastric bypass

## Abstract

**Background:**

It is imperative to assess the results of revisional procedures following Sleeve Gastrectomy (SG), given the substantially growing population of patients who experience weight regain within a few years after undergoing this procedure.

**Objective:**

Examine the comparative effectiveness of the Single Anastomosis Duodeno-Ileal Bypass (SADI-S) and the One Anastomosis Gastric Bypass (OAGB-MGB) as revisional procedures, with respect to their impact on weight loss, resolution of comorbidities, incidence of complications, and rates of reoperation in patients who had weight regain after SG with up to or more than 5 years of follow-up.

**Setting:**

Hamad General Hospital, Academic tertiary referral center, Qatar.

**Methods:**

This study retrospectively analyzed a database of patients who underwent the Single Anastomosis Duodeno-Ileal Switch (SADI-S) or the One Anastomosis Gastric Bypass - Mini Gastric Bypass- (OAGB-MGB) as revisional procedures for weight recidivism after a primary Laparoscopic Sleeve Gastrectomy (LSG). The follow-up period was at least 5 years, during which the impact of both procedures on weight loss, comorbidities, nutritional deficiencies, complications, and outcomes were compared.

**Results:**

The study comprised 91 patients, with 42 and 49 in the SADI-S and OAGB-MGB groups, respectively. Significant weight loss (measured by total weight loss percentage, TWL%) was observed at the 5-year follow-up for the SADI-S group compared to the OAGB-MGB group (30.0 ± 18.4 vs. 19.4 ± 16.3, *p* = 0.008). Remission of comorbidities, specifically diabetes mellitus and hypertension, was more prevalent in the SADI-S group. Notably, the OAGB-MGB group had a higher incidence of complications (28.6% vs. 21.42%) and reoperations (5 patients vs. 1 in the SADI-S group). No mortality events were reported in either group.

**Conclusion:**

While both the OAGB-MGB and SADI-S have demonstrated efficacy as revisional procedures for weight regain following SG, the SADI-S exhibits superior outcomes compared to the OAGB-MGB with regard to weight loss, resolution of comorbidities, complication rates, and reoperation rates.

As per the International Surgery of Obesity and Metabolic Disorders (IFSO), SG has emerged as the most frequently performed bariatric surgery in 2016 [1, 2]. Nevertheless, a significant percentage of patients, ranging from 10 to 50%, are subject to weight regain as per the 10-year follow-up studies [3–5].

Studies with a 10-year follow-up have reported an incidence of 18 to 36% need for revisional procedures after SG due to inadequate weight loss, weight regain, or complications [4, 5].

There are various revisional procedures available after sleeve gastrectomy (SG), such as conversion to Roux-en-Y gastric bypass (RYGB) [6], one anastomosis gastric bypass (OAGB-MGB) [7], Single anastomosis duodeno-ileal bypass (SADI-S), and duodenal switch (DS) [8–10]. The assessment of revisional procedures following SG is imperative, given that a considerable and consistent proportion of patients may require a conversion in the future.

The SADI-S and the OAGB-MGB have been proposed as technically simpler yet equally effective alternatives to the Roux-en-Y procedures, namely the biliopancreatic diversion with duodenal switch (BPD-DS) and the Roux-en-Y Gastric bypass (RYGB), respectively [11, 12].

In our prior publication, our group has reported on the short-term comparative efficacy and outcome of SADI-S to OAGB-MGB, using the same database [13].

There are two reasons for comparing the two procedures. Firstly, both the one anastomosis gastric bypass (OAGB-MGB) and single anastomosis duodeno-ileal bypass with sleeve gastrectomy (SADI-S) are considered as hypo-absorptive methods and have similar effects in terms of weight loss. Secondly, the shared loop configuration and the nature of having only one anastomosis make it reasonable to perform a direct comparison between the two procedures [14].

The objective of this study is to examine the comparative effectiveness of the SADI-S and the OAGB-MGB as revisional procedures, with respect to their impact on weight loss, resolution of comorbidities, incidence of complications, and rates of reoperation in patients who had weight regain after SG with up to or more than 5 years of follow-up.

## Methods

A retrospective observational study of a prospectively collected data from the electronic medical records at Hamad General Hospital, Qatar. The inclusion criteria were patients who underwent SADI-S or OAGB-MGB as revisional procedure for weight regain post sleeve gastrectomy in the time between January 2016 and August 2017 and completed at least 5 years post procedure. The exclusion criteria were patients who underwent SADI-S or OAGB-MGB as a primary procedure, or as a planned secondary procedure and patients with less than 5 years of follow-up after surgery.

Primary outcome: weight loss 5 years after the revisional procedure. Weight loss was measured and presented by multiple parameters including BMI, excess weight loss percentage (EWL%), and total weight loss percentage (TWL%).

Secondary outcome: metabolic profile parameters, obesity-related comorbidities mainly type 2 diabetes mellitus (T2D), hypertension, dyslipidemia, in addition to blood markers and postoperative complications.

Remission of T2D was defined as hemoglobin A1c (A1C) < 6.5 and/or free blood glucose < 100 mg/dl, and remission of hypertension was defined as normotensive with blood pressure < 130/90 mmHg off medications [15].

The blood markers evaluated were hemoglobin (Hb), total protein level, albumin, lipid profile markers (triglycerides, cholesterol, LDL, HDL), iron, zinc, INR, vitamin B12, and vitamin D.

### Preoperative evaluation

Prior to the surgical procedure, all patients underwent a comprehensive preoperative assessment involving extensive laboratory investigations and nutritional evaluation. As part of our standard protocol for patients being considered for revisional bariatric procedures, Esophagogastroduodenoscopy (EGD) and Upper gastrointestinal series (Barium meal) were performed to assess the upper gastrointestinal tract (Esophagus and sleeved stomach).

### Surgical procedures

#### SADI-S procedure

All patients included in this study had previously undergone sleeve gastrectomy, and thus, no re-sleeve or trimming procedures were performed on the existing gastric sleeve. Retroduodenal dissection was conducted using an ultrasonic energy device, primarily until the level of the gastroduodenal artery, with the objective of obtaining a duodenal stump located 2–4 cm distal to the pyloric ring. Additionally, the peritoneum covering the hepatoduodenal ligament at the superior border of the duodenum was incised to facilitate the creation of a retroduodenal tunnel for the stapler. The duodenum was divided at this level using a linear stapler measuring 60–3.5 mm. Subsequently, the ileocecal junction was identified, and a segment of the ileum measuring 250–300 cm was measured and brought up to be anastomosed with the proximal end of the divided duodenum, employing a double-layered duodeno-ileostomy technique utilizing a 3/0 barbed suture. The majority of patients in this study had a 300 cm ileal loop, while only five patients had a loop of 250 cm. A methylene blue leak test was routinely performed in all cases, and no drains were employed. Clear oral fluids were allowed 6 h after surgery, and patients were discharged on either the first or second postoperative day.

#### OAGB-MGB procedure

The previously sleeve stomach was typically divided at the level of the incisura angularis to create an elongated gastric pouch. The duodeno-jejunal junction was identified, and a jejunal loop measuring approximately 150–200 cm was ascended in an antecolic fashion to establish a side-to-side gastro-jejunal anastomosis using a linear stapler. The enterotomy was closed using a double-layered technique with a 3/0 barbed suture. As a routine practice, we utilized a 150 cm jejunal loop starting from the duodeno-jejunal junction, although four patients had a 180 cm loop and three patients had a 200 cm jejunal loop. Similarly, a methylene blue leak test was performed in all cases, and no drains were utilized. Patients were allowed to start clear oral fluids 6 h after surgery and were typically discharged on either the first or second postoperative day.

All patients who underwent the SADI-S or OAGB-MGB procedures were provided with the same postoperative bariatric protocol, which encompassed dietary instructions, protein supplements, multivitamins, proton pump inhibitors (PPIs), and scheduled outpatient follow-up appointments.

### Statistical methods

Descriptive statistics in the form of mean and standard deviation for continuous variables and frequency with percentages for categorical variables were calculated. Student paired *t* tests or Wilcoxon signed tests as appropriate were applied to see statistically significant differences within the SADI-S and OAGB-MGB groups separately for all continuous variables. Chi-square tests were applied to see association of categorical variables with the two groups (SADI-S vs OAGB-MGB). Mean differences of post clinical variables were compared using unpaired student *t* tests or Wilcoxon rank-sum tests as appropriate between the SADI-S and OAGB-MGB groups. *p* value 0.05 (two tailed) was considered as statistically significant level. SPSS 29.0 statistical package was used for the analysis.

### Ethics

Ethical approval for this study was obtained from the Institutional Review Board (IRB) at Hamad Medical Corporation (HMC), Medical Research center (MRC), Doha, Qatar (MRC-01-19-335).

## Results

### Preoperative characteristics

The study population were analyzed, which included 91 patients who underwent either SADI-S (*n* = 42) or OAGB-MGB (*n* = 49) as revisional procedures for weight regain post sleeve gastrectomy and had a follow-up of at least 5 years. The mean age of both study groups was approximately 38 years, with a higher proportion of females. The mean pre-SG weight was 133 ± 29.1 kg in the OAGB-MGB group and 141.5 ± 27.8 kg in the SADI-S group. The mean pre-SG BMI was 52 ± 11 kg/m^2^ for the OAGB-MGB group and 50 ± 8 kg/m^2^ for the SADI-S group. The mean pre-revisional procedure BMI was 43.0 ± 6.8 for the OAGB-MGB group, and 45.9 ± 10.3 in the SADI-S group.

### OAGB-MGB

Table 1 provides a comprehensive overview of the demographics, anthropometric measurements, and complications observed in patients who underwent the OAGB-MGB procedure. The sample consisted of individuals with a male-to-female ratio of 1:6 and an average age of 38 ± 9 years. The mean preoperative BMI was 43.0 ± 6.8 kg/m^2^, which was significantly reduced to 35.6 ± 7.0 kg/m^2^ at 5 years after revisional surgery. Table 2 presents a comparison of pre- and post-procedure blood marker levels. The laboratory tests showed a statistically significant improvement in the A1C, serum cholesterol, LDL, and HDL levels. Notably, the OAGB-MGB procedure resulted in a reduction Hb level 5 years after surgery (*p* = 0.02).

**Table 1:** Demographic, anthropometric, complications, and outcome data of patients underwent OABG as revisional procedure for weight regain (*n* = 49)

Gender (M:F)	7:42
Age (mean ± standard deviation)	37.83 ± 9.36
No. of years follow-up	3.8 ± 1.4
Weight	
• Before LSG	133.5 ± 29.1
• Before revisional procedure	113.3 ± 20.2
• 5 Years after revision	93.8 ± 19 (*p* value < 0.001)
BMI	
• Before revisional procedure	43.0 ± 6.8
• 5 Years after revision	35.6 ± 7.0 (*p* value < 0.001)
TWL% 5 years post-revisional procedure	19.4 ± 16.3
EWL% post-revisional procedure	
• 5 Years after revision	50.9 ± 30.6
Postoperative complications (n)	
• Staple line leak	1 (RYGB)
• Anastomotic ulcer	3
• Bile reflux	3 (2 RYGB)
• Denovo GERD	3
• Nutritional deficiency	1
• Revisional surgery	2 (SADI)
• Mortality	0

OABG: One Anastomosis Gastric Bypass; LSG: laparoscopic sleeve gastrectomy; BMI: Body Mass Index; TWL%: total weight loss percentage; EWL %: excess weight loss percentage; GERD: gastroesophageal reflux disease; RYGB: Roux-en-Y Gastric bypass; SADI: Single Anastomosis Duodeno-Ileostomy

**Table 2:** Comparison of blood markers level before and after OABG as revisional procedure for weight regain (*n* = 49)

Parameters	Before OABG (Mean ± SD)	5 Years after OABG (Mean ± SD)	*p* value
Hemoglobin A1C (HBA1C)	5.5 ± 0.9	5.3 ± 0.4	0.01
Protein	69.2 ± 4.6	69.2 ± 4.8	1.0
Hemoglobin (Hb)	12.1 ± 1.5	11.5 ± 1.9	0.02
Albumin	36.4 ± 3.1	36.5 ± 4.7	0.90
Zinc	11.6 ± 2.9	11.6 ± 2.2	0.97
Vitamin B12	250.6 ± 86.6	237.8 ± 101.5	0.46
International Normalized Ratio (INR)	0.99 ± 0.04	1.0 ± 0.03	0.13
Vitamin D	17.80 ± 9.95	19.2 ± 11.7	0.31
Triglycerides	1.2 ± 0.5	0.9 ± 0.4	0.03
Cholesterol	5.1 ± 0.9	4.7 ± 0.9	0.02
High-density lipoprotein (HDL)	1.5 ± 0.5	1.6 ± 0.4	0.02
Low-density lipoprotein (LDL)	3.2 ± 0.9	2.5 ± 0.7	0.001
Iron	12.1 ± 5.9	12.0 ± 6.4	0.95

*SD* standard deviation

### SADI-S

Table 3 displays the demographic, anthropometric, and complication data of patients who received the SADI-S procedure. The male-to-female ratio observed was 2:5. Notably, there was a marked reduction in BMI 5 years after undergoing SADI-S, from an average of 45.9 ± 10.3 to 33.7 ± 5.8 (*p* value < 0.001). Furthermore, the percentage of TWL% and EWL% exhibited significant increases at 5 years post operation. Table 4 presents a comparative assessment of blood marker levels before and after undergoing the SADI-S procedure. A statistically significant reductions were observed in the serum levels of A1c and lipid profile (triglyceride, cholesterol, HDL and LDL). Moreover, the serum level of Hb was also reduced (*p* value 0.005).

**Table 3:** Demographic, anthropometric, complications, and outcome data of patients underwent SADI as revisional procedure for weight regain (*n* = 42)

Gender (M:F)	12:30
Age (mean ± standard deviation)	38.0 ± 9.0
No. of years follow-up	5.0 ± 1.4
Weight	
• Before LSG	141.5 ± 27.8
• Before revisional procedure	121.6 ± 24.4
• 5 Years after revision	91.4 ± 16.1 (*p* value < 0.001)
BMI	
• Before revisional procedure	45.9 ± 10.3
• 5 Years after revision	33.7 ± 5.8 (*p* value < 0.001)
TWL% 5 years post-revisional procedure	30.0 ± 18.4
EWL% 5 years post-revisional procedure	66.2 ± 21.7 (*p* value < 0.001)
Postoperative complications (n)	
Abdominal collection	1
Steatorrhea	6
Nutritional deficiency	1
Intractable GERD	1 (RYGB)
Mortality	0

*SADI* Single Anastomosis Duodeno-Ileostomy; *LSG* laparoscopic sleeve gastrectomy; *BMI* Body Mass Index; *TWL*% total weight loss percentage; *EWL*% excess weight loss percentage

**Table 4:** Comparison of blood markers level before and after SADI-S as revisional procedure for weight regain (*n* = 42)

Blood markers	Before SADI (Mean ± SD)	5 years after SADI (Mean ± SD)	*p* value
HemoglobinA1C (HBA1C)	5.7 ± 0.9	5.2 ± 0.6	0.001
Total Protein	66.2 ± 8.6	66.9 ± 8.3	0.62
Hemoglobin	12.6 ± 1.7	11.7 ± 2.3	0.005
Albumin	35.7 ± 3.4	36.4 ± 4.0	0.29
Zinc	11.1 ± 2.3	11.3 ± 3.8	0.70
Vitamin B12	282.2 ± 243.7	425.5 ± 283.8	0.002
International Normalized Ratio (INR)	1.0 ± 0.05	1.1 ± 0.06	0.13
Vitamin D	15.42 ± 5.75	11.76 ± 5.32	0.006
Triglycerides	1.03 ± 0.3	0.8 ± 0.4	0.006
Cholesterol	5.0 ± 0.9	4.1 ± 0.8	0.001
High-density lipoprotein (HDL)	1.4 ± 0.3	1.5 ± 0.3	0.03
Low-density lipoprotein (LDL)	3.0 ± 0.9	2.1 ± 0.7	0.001
Iron	11.8 ± 6.2	14.4 ± 11.5	0.14

*SD* standard deviation

### SADI-S versus OAGB-MGB

Table 5 displays the outcomes of a comparative analysis conducted on the two revisional procedures. The mean duration between the primary surgery (SG) and the revisional procedure was between 24 and 48 months for both interventions. The preoperative weight and BMI were equivalent for both groups. However, the weight assessment variables measured 5 years post surgery, including the difference in BMI, TWL%, and EWL%, all showed statistically significant differences in favor of the SADI-S procedure. Table 6 presents the findings of a comparative assessment of blood biomarkers between the two procedures. At 5 years of follow-up, patients who underwent the SADI-S procedure demonstrated significant enhancements in serum lipids profile, including triglycerides, cholesterol, LDL, and HDL, when compared to those who underwent OAGB-MGB. Conversely, there were no statistically significant differences in A1C values, serum protein, serum albumin, INR, serum zinc, and Hb levels between the two groups, indicating comparable outcomes.

**Table 5:** Comparative analysis of anthropometric measures before and 5 years after revisional surgeries (OAGB vs. SADI)

Parameters	OAGB Mean ± SD	SADI Mean ± SD	*p* value
Weight before LSG surgery	133 ± 27.82	139.06 ± 27.19	0.289
Preoperative Weight	114.18 ± 21.08	119.32 ± 23.90	0.268
Preoperative BMI	43.58 ± 7.38	43.90 ± 7.37	0.832
EW	48.41 ± 18.29	51.40 ± 20.80	0.458
Δ BMI	7.4 ± 5.7	12.2 ± 8.9	0.006
TWL %	19.4 ± 16.3	30.0 ± 18.4	0.008
EWL %	50.9 ± 30.6	66.2 ± 21.7	0.01

**Table 6:** Comparative analysis of blood marker levels before and 5 years after revisional surgeries (OAGB vs. SADI)

Parameters	OAGB Mean ± SD	SADI Mean ± SD	*p* value
Preoperative HBA1C	5.48 ± 0.68	5.77 ± 1.06	0.151
HBA1C 5 years post revision	5.3 ± 0.4	5.2 ± 0.6	0.51
Preoperative Protein	69.43 ± 4.40	68.71 ± 5.38	0.521
Protein 5 years post revision	69.2 ± 4.8	67 ± 8.0	0.15
Preoperative Hb	12.07 ± 1.50	12.63 ± 1.69	0.100
Hb 5 years post revision	11.5 ± 1.86	11.7 ± 2.3	11.7 ± 2.3
Preoperative Albumin	36.14 ± 3.12	35.54 ± 3.49	0.216
Albumin 5 years post revision	36.5 ± 4.7	36.4 ± 4.0	0.90
Preoperative zinc	12.14 ± 1.80	12.15 ± 2.06	0.998
Zinc 5 years post revision	11.6 ± 2.2	11.2 ± 3.5	0.61
Preoperative Vitamin B12	248.28 ± 84.54	310.51 ± 267.72	0.160
Vitamin B12 5 years post revision	239 ± 99.9	421 ± 282	0.001
Preoperative INR	1 ± 0.04	1 ± 0.07	0.857
INR 5 years post revision	1.0 ± 0.03	1.01 ± 0.05	0.34
Preoperative Vitamin D	17.46 ± 10.24	13.84 ± 5.97	0.078
Vitamin D 5 years post revision	22.8 ± 11.0	19.9 ± 8.9	0.18
Preoperative triglycerides	1.16 ± 0.54	1.13 ± 0.33	0.833
Triglycerides 5 years post revision	1.0 ± 0.4	0.8 ± 0.4	0.07
Preoperative cholesterol	5.13 ± 1.01	5.18 ± .86	0.818
Cholesterol 5 years post revision	4.7 ± 0.9	4.1 ± 0.8	0.004
Preoperative HDL	1.45 ± 0.49	1.43 ± 0.25	0.831
HDL 5 years post revision	1.6 ± 0.4	1.5 ± 0.3	0.03
Preoperative LDL	3.18 ± 0.94	3.24 ± 0.76	0.801
LDL 5 years post revision	2.4 ± 0.7	2.1 ± 0.7	0.05
Preoperative Iron	12.46 ± 6.02	10.77 ± 5.95	0.229
Iron 5 years post revision	11.8 ± 6.4	13.8 ± 10.9	0.33

OABG: One Anastomosis Gastric Bypass; SADI: Single Anastomosis Duodeno-Ileostomy; LSG: laparoscopic sleeve gastrectomy; SD: standard deviation; BMI: Body Mass Index; EW:?? WL: weight loss; TWL%: total weight loss percentage; EWL %: excess weight loss percentage; Hb: Hemoglobin; INR: International Normalized Ratio; HDL: High-density lipoprotein; LDL: Low-density lipoprotein

### Nutritional deficiency

Upon examination of blood markers indicative of nutritional deficiency 5 years following surgery, including Hb, serum protein, serum albumin, INR, and vitamin B12, there was no statistically significant difference observed between the two procedures. However, it is noteworthy that over-replacement of vitamin B12 was observed in the SADI-S procedure.

### Comorbidities

In the SADI-S group, 6 out of 8 patients (75%) who had T2D experienced resolution, while the remaining two patients demonstrated a reduction in their anti-diabetic medications. This outcome is evidenced by a statistically significant reduction in abnormal A1C values (*p* value = 0.001). Two patients out of four (50%) had resolution of hypertension and stopped the anti-hypertensive medications. In comparison to the OAGB-MGB group, 3 patients out of 6 (50%) had resolution of T2D and one patient out of five had hypertension became normotensive and discontinued his anti-hypertensive medications.

### Complications

In the OAGB-MGB group, 14 out of 49 patients (28.6%) experienced complications, with 5 cases (10.2%) requiring conversion to another procedure. Specifically, two patients required conversion to Roux-en-Y gastric bypass (RYGB) due to severe bile reflux, one patient required conversion to RYGB due to leak, and two patients were converted to SADI-S due to weight regain. Three cases developed marginal anastomotic ulcer and were treated medically, while an additional three patients developed de novo acid reflux (GERD). Within the SADI-S group, 9 out of 42 patients (21.42%) experienced complications following surgery. One patient required conversion to Roux-en-Y gastric bypass (RYGB) due to severe acid reflux that was unresponsive to medical treatment. Six cases of postoperative steatorrhea were observed, which all resolved within 3 to 6 months. One patient developed an intra-abdominal collection that was successfully managed through CT-guided drainage. Nutritional deficiency necessitated hospital re-admission for total parenteral nutrition (TPN) in two patients, with one case occurring in each group. No mortality cases were reported in both groups.

## Discussion

In 2020, we have reported on the short-term efficacy and outcomes of the two procedures as revisional options following sleeve gastrectomy. The conclusion drawn was that both procedures had similar outcomes [13]. In the present study, we aimed to evaluate the outcomes of both procedures after 5 years from the time of revision. Our analysis indicates that while both procedures are effective for addressing weight recidivism following sleeve gastrectomy, the SADI-S procedure demonstrated statistically significant superior outcomes compared to the OAGB-MGB procedure in terms of weight reduction, resolution of comorbidities, complications rate, and need for reoperation.

The current study has revealed that the SADI-S procedure leads to a more substantial reduction in weight when compared to the OAGB-MGB procedure. The SADI-S group exhibited a total weight loss percentage (TWL%) of 30.0 ± 18.4, whereas the OAGB-MGB group had a TWL% of 19.4 ± 16.3. Moreover, the excess weight loss percentage (EWL%) for the SADI-S group was 66.2 ± 21.7, which was significantly higher than the EWL% of 50.9 ± 30.6 for the OAGB-MGB group. Furthermore, the SADI-S group experienced a drop in BMI of 12.2 ± 8.9, which was considerably greater than the drop of 7.4 ± 5.7 BMI observed in the OAGB-MGB group (*p* value of 0.006). These findings corroborate prior research that has suggested the SADI-S procedure is an effective means of achieving significant weight loss, as demonstrated by Dijkhorst et al. [16] who reported successful outcomes using the SADI-S as a revisional bariatric procedure [16–18]. Our own study aligns with previous research, with two prior studies also reporting an EWL% of 66.2 ± 21.7 following the SADI-S procedure over a 5-year period. Taken together, these results provide further support for the use of the SADI-S procedure as a reliable option for revisional bariatric surgery to achieve sustained weight loss.

The second outcome which was analyzed in our study was the resolution of comorbidities. Although both procedures had achieved a positive impact on the resolution of comorbidities (mainly T2D, HTN, and dyslipidemia), but the SADI-S had shown a far higher rate of resolution of comorbidities 5 years after surgery. In a study by Sanchez-Pernaute et al. [9], a 52% remission rate of type 2 diabetes (T2D) was reported after a 5-year follow-up. On the other hand, Zaveri et al. [18] found an 81% remission rate of T2D over a 4-year follow-up period post SADI-S [9, 18].

This study evaluated the incidence of complications and the rate of reoperations following the two revisional procedures. The results revealed a significantly higher occurrence of both complications and reoperation in the OAGB-MGB group. Specifically, five patients in the OAGB-MGB group necessitated conversion to a different procedure due to issues such as leak, bile reflux, and weight regain, whereas only one patient in the SADI-S group was converted to RYGB owing to intractable GERD.

In the SADI-S cohort, diarrhea/steatorrhea was the foremost complication observed in 6 patients (14%), a finding that has been corroborated in multiple studies [19]. While not as severe as those in patients who underwent BPD, such complications were frequently encountered following the SADI-S procedure [19, 20]. Nonetheless, our study determined that this complication was transient and resolved spontaneously in all patients within a period of 3 to 6 months. In contrast, no similar complications were noted among patients in the OAGB-MGB cohort.

When comparing nutritional deficiencies after SADI, studies have generally reported a higher percentage of deficiencies than what was observed in our patients [21–23]. It is possible that this discrepancy may be attributed to differences in the length of the common channel. Previous studies included cases with a common channel length of 200 cm, while the common channel length for all our patients was 250 to 300 cm. This study observed a limited occurrence of severe malnutrition and hypoalbuminemia within the initial year after surgery, with only two patients (one from each group) affected. These cases were managed through hospitalization and total parenteral nutrition (TPN) for a period of 2 weeks.

### Limitations

Our study is subject to several limitations. Notably, its retrospective design, despite the use of prospectively collected data from electronic medical records. Furthermore, there is a dearth of information regarding quality of life following revisional procedures. However, our study boasts several strengths, including a comparatively larger sample size for revisional procedures than previous studies and a longer follow-up duration of 5 years post surgery.

## Conclusions

In summary, the SADI-S procedure exhibits a superior outcome as a revisional option for weight recidivism after SG compared to the OAGB-MGB in terms of weight loss, resolution of comorbidities, and rates of complications and reoperation. Nonetheless, the OAGB-MGB still serves as an effective and safe alternative for patients experiencing weight regain following SG.
